# Mr. Brenan, on Pulmonary Polypi

**Published:** 1802-10-01

**Authors:** James Brenan

**Affiliations:** Brompton, Chatham


### *To the Editors of the Medical and Phyfical Journal.*

GENTLEMEN,

ON perufing the laft Number of your very ufeful Journal, my attention was particularly attracted by the defcription of a Pulmonary Polypus, related by Doctor Eric Acharius; and this cafe called to my recollection a fimilar one that had offered itfelf to my notice fome time fince, when I was Firft Affiftant Surgeon on board his Majefty's hofpital fhip Argonaut, at Chatham. The remark that Dr. Warren was the only perfon who had recorded an inftance in all refpects correfpondent, has induced me to offer the following to your notice, with a requeft that you will publifh it if you deem it worthy a place in your Mifcellany.

Mr. William Afhburner, mafter's mate of his Majefty's fhip Lion, about forty years of age, was received into the hofpital on the 7th of October, 1800, labouring under typhus fever. The furgeon of the Lion had not fent with the patient any precife ftatement of his cafe; but I learnt the following particulars from an officer belonging to that fhip, who was received into the Argonaut at the fame time with Mr. Afhburner. He had been on the Mediterranean ftation three years, and during that time had been frequently afflicted with fcurvy; His general health was very indifferent; his conftitution was impaired by a too liberal indulgence in fpirituous liquors, and he had been ill fome days previous to his removal from the Lion.

Lion. I ſaw him in an hour after he came on board; he was in a low delirium; a bliſter was immediately applied between his ſhoulders, and the Anguſtura bark, with aromatic confection, was copiouſly exhibited. He was ordered to drink a pint of Port wine within the next twenty-four hours. The medicine ſat well on the ſtomach; but the wine uniformly excited a vomiting and bleeding at the noſe. This was as diſtreſſing as remarkable, for his debility being ſo great, every drop of blood effuſed was a loſs of a valuable conſideration. On changing the red wine for Mountain, and the Anguſtura for Cinchona, with vitrolic acid, theſe unpleaſant effects ceaſed. He drank a bottle of this wine daily, during the remainder of his illneſs. The delirium continued, and he became gradually lower until the 13th, when the nurſe informed me, that he had coughed up a knot of ſmall worms, and that he had, on the ſame morning, frequently brought up others, which ſhe had thoughtleſsly thrown away. On this day he died. Before this expectorated ſubſtance was waſhed, it had much the appearance of the tape worm; but after cleaning it from a dirty looking mucus, I obſerved it divided into numerous branches, which again ramified into ſmaller twigs, forked like the claws of a green crab. From its appearance, and the manner in which it was expectorated, I have no doubt of its being a pulmonary polypus. I have preſerved it in ſpirits, but it begins to change its colour, which at firſt was very white, and appears to rather diſſolve in the ſpirits. As theſe polypi are not common, and as it may be of more utility when diſplayed amongſt a collection of rarities than it can be in the narrow circle of my communication, I beg to offer it to you as a preſent, if you think it may merit your acceptance.

I am, &c.

*Brompton, Chatham,*

*Sept.* 10, 1802.

JAMES BRENAN.

